# Cytomegalovirus IgM Seroprevalence among Women of Reproductive Age in the United States

**DOI:** 10.1371/journal.pone.0151996

**Published:** 2016-03-18

**Authors:** Chengbin Wang, Sheila C. Dollard, Minal M. Amin, Stephanie R. Bialek

**Affiliations:** Centers for Disease Control and Prevention, Atlanta, Georgia, United States of America; University of St Andrews, UNITED KINGDOM

## Abstract

Cytomegalovirus (CMV) IgM indicates recent active CMV infection. CMV IgM seroprevalence is a useful marker for prevalence of transmission. Using data from the National Health and Nutrition Examination Survey (NHANES) III 1988–1994, we present estimates of CMV IgM prevalence by race/ethnicity, provide a comparison of IgM seroprevalence among all women and among CMV IgG positive women, and explore factors possibly associated with IgM seroprevalence, including socioeconomic status and exposure to young children. There was no difference in IgM seroprevalence by race/ethnicity among all women (3.1%, 2.2%, and 1.6% for non-Hispanic white, non-Hispanic black and Mexican American, respectively; P = 0.11). CMV IgM seroprevalence decreased significantly with increasing age in non-Hispanic black women (P<0.001 for trend) and marginally among Mexican American women (P = 0.07), while no apparent trend with age was seen in non-Hispanic white women (P = 0.99). Among 4001 IgG+ women, 118 were IgM+, resulting in 4.9% IgM seroprevalence. In IgG+ women, IgM seroprevalence varied significantly by age (5.3%, 7.3%, and 3.7% for women of 12–19, 20–29, and 30–49 years; P = 0.04) and race/ethnicity (6.1%, 2.7%, and 2.0% for non-Hispanic white, non-Hispanic black, and Mexican American; P<0.001). The factors reported associated with IgG seroprevalence were not associated with IgM seroprevalence. The patterns of CMV IgM seroprevalence by age, race/ethnicity, and IgG serostatus may help understanding the epidemiology of congenital CMV infection as a consequence of vertical transmission and are useful for identifying target populations for intervention to reduce CMV transmission.

## Introduction

Cytomegalovirus (CMV) is a common human viral pathogen that typically causes minimal, if any, symptoms in immunocompetent individuals.[1] However, CMV infection can cause severe outcomes and even death in immunocompromised individuals and infants infected in utero.[2–4] Active CMV infection during pregnancy is the leading viral cause of birth defects and developmental disabilities in developed countries.[2]

An individual develops lifetime CMV IgG seropositivity (IgG+) after primary infection (the first infection in life), after which CMV establishes latency with intermittent reactivation. CMV IgG+ individuals can be reinfected with another strain of CMV. CMV IgM can be produced after primary infection and after non-primary infection (reactivation or reinfection).[5] It typically is detectable for only a few months,[6] and indicates recent active CMV infection. The transiency of IgM makes CMV IgM seroprevalence rates a useful marker for prevalence of transmission in a population at the time of testing.

In the U.S., CMV IgM seroprevalence in the general population have been briefly described among women aged 12–49 years from the National Health and Nutrition Examination Survey (NHANES) III 1988–1994.[7] The lack of temporal changes in CMV IgG seroprevalence from 1988–1994 to 1999–2004 [8] suggests that the factors associated with CMV transmission have remained fairly consistent over time and that findings on IgM seroprevalence from NHANES III are still informative for understanding the epidemiology of acute CMV infection and risk of transmission. We expand on the previous analysis of IgM seroprevalence among US women [7] by presenting estimates by race/ethnicity and by re-categorized age groups. We also present a comparison of IgM seropositivity by race/ethnicity and age among all women and among CMV IgG positive women to investigate whether future assessments of IgM seropositivity could be conducted using a less expensive, more streamlined approach. In addition, we explore factors possibly associated with IgM seroprevalence, including those previously identified as being associated with CMV IgG seroprevalence such as socioeconomic status and exposure to young children.[5]

## Materials and Methods

Publically accessible data on CMV IgG and IgM of NHANES III 1998–1994 were analyzed and IgM was only tested on women aged 12–49 years of age while IgG data available for all NHANES III participants.[9] NHANES III was conducted by the Centers for Disease Control and Prevention from 1988 to 1994 and was a complex, stratified, multistage probability cluster sample of the noninstitutionalized civilian of the United States. The detailed methodology and response rates of NHANES III are publically accessible.[10] In contrast to a previously published analysis of all female NHANES participants,[7] only women of three racial/ethnic groups (non-Hispanic white, non-Hispanic black and Mexican American) were included in the current analysis in order to provide estimates by racial/ethnic groups that were consistent with previous analyses of CMV IgG seroprevalence.[5, 8] NHANES methods for serum sample selection and laboratory testing for CMV IgG and IgM have been reported previously.[5, 7]

Age was categorized as 12–19, 20–29, and 30–49 years with varied intervals to ensure that there were at least 5 individuals in each subgroup as fewer subjects were IgM+ in those aged ≥30 years. Nationally representative estimates of IgM seroprevalence were calculated using the same modified weights as in the prior report [7] after accounting for the proportion of available serum samples by age groups and race/ethnicity with SAS Survey Procedures (SAS Inc., Cary, NC). Risk factors associated with CMV IgG seroprevalence such as socioeconomic status and exposure to young children were categorized as reported previously [5] and examined for their association with CMV IgM seroprevalence in all and CMV IgG+ women. Home exposure to young children was defined as reporting having at least one child aged ≤6 years at home.

## Results

**Fig 1:**
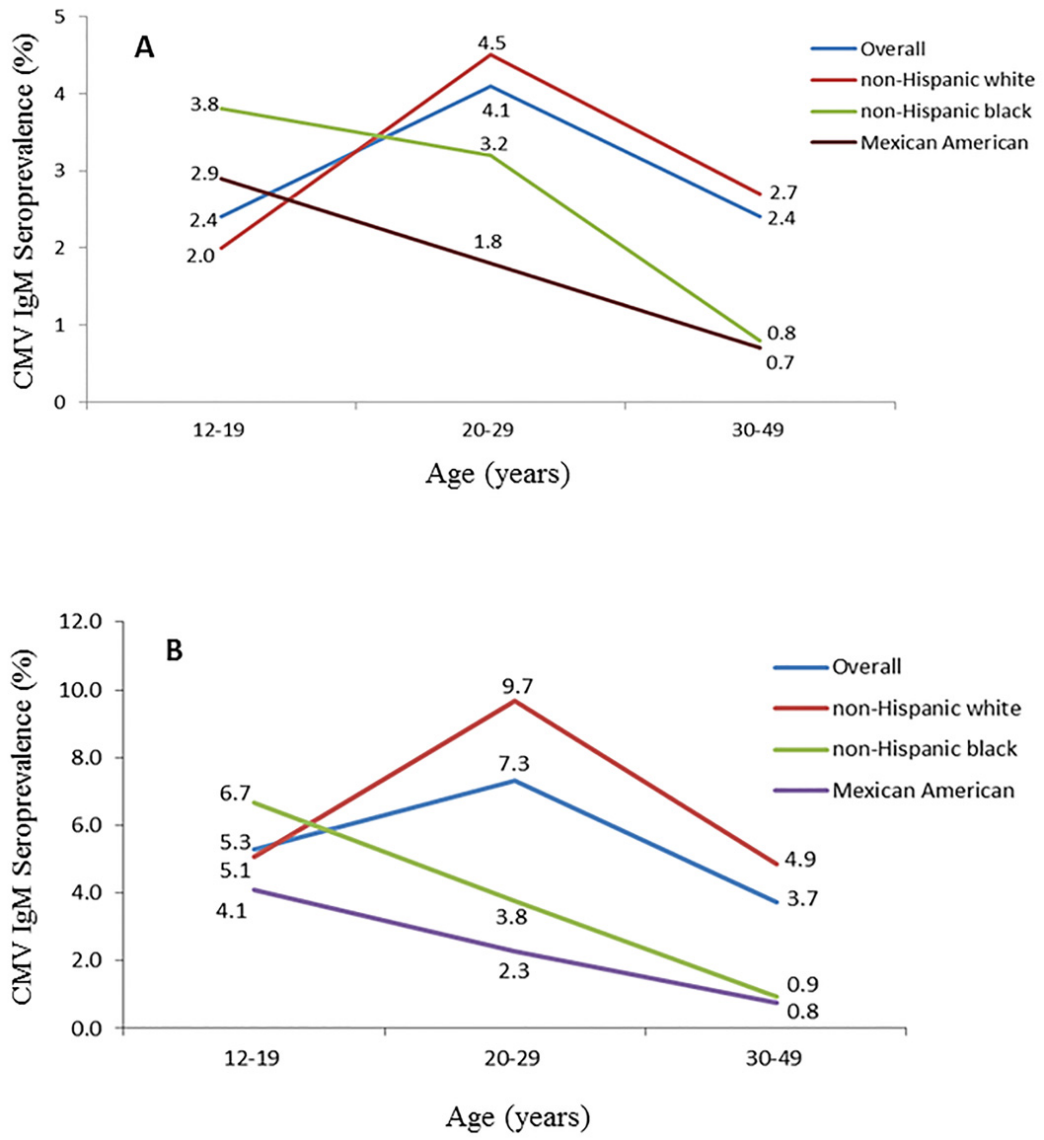
Cytomegalovirus IgM seroprevalence among All Women (Panel A) and IgG+ Women only (Panel B) by Age and Racial/ethnic Group, NHANES III, 1988–1994.

Among the 5714 women included in the analysis, approximately half were aged 30–49 years (2695, 47.2%) and similar proportions were aged 12–19 years and 20–29 years (1445 and 1574, 25.3% and 27.6%, respectively). Non-Hispanic white, non-Hispanic black and Mexican American participants each represented about one third of the study population (1825, 2013, and 1876; 31.9%, 35.2% and 32.8%, respectively). The majority of participants were IgG+ (4001, 70%). A total of 121 women were CMV IgM+ for an overall prevalence estimate of 2.8% [95% confidence interval (CI): 2.1–3.6%] among these three racial/ethnic groups. Overall, CMV IgM seroprevalence did not differ significantly by race/ethnicity (3.1%, 2.2%, and 1.6% for non-Hispanic white, non-Hispanic black and Mexican American, respectively; P = 0.11). When stratified by age, there was no difference by race/ethnicity among women aged 12–19 years (P = 0.38), but non-Hispanic white women had higher IgM seroprevalence compared to Mexican American women at 20–29 years of age (4.5% vs. 1.8%, P = 0.02), and higher IgM seroprevalence compared to non-Hispanic black and Mexican American women at age of 30–49 years (2.7% vs. 0.8% and 0.7% for non-Hispanic white, non-Hispanic black, and Mexican American, respectively; P = 0.03 and 0.01). IgM seroprevalence marginally varied by age with the highest IgM seroprevalence among those aged 20–29 years and lower seroprevalence among those aged 12–19 or 30–49 years (4.1%, 2.4% and 2.4%, respectively; P = 0.05). The patterns of CMV IgM seroprevalence with age differed by race/ethnicity. CMV IgM seroprevalence decreased significantly with increasing age in non-Hispanic black women (P<0.001 for trend) and marginally among Mexican American women (P = 0.07), while no apparent trend with age was seen in non-Hispanic white women (P = 0.99) (Fig 1, Panel A).

Among the 121 CMV IgM+ women, 118 (97.5%) were CMV IgG+ and three (2.5%) were IgG-. Among the 4001 CMV IgG+ women, overall IgM seroprevalence was 4.9% (95% CI: 3.4–6.4%), and CMV IgM seroprevalence varied significantly by race/ethnicity (6.1% vs. 2.7% and 2.0% for non-Hispanic white vs. non-Hispanic black and Mexican American, respectively; P = 0.002 and 0.005). When stratified by age, there was no difference by race/ethnicity among women aged 12–19 years (P = 0.58), but non-Hispanic white women had higher IgM seroprevalence than women of other two racial/ethnic groups at age of 20–29 years (9.7% vs. 3.8% and 2.3% for non-Hispanic white, non-Hispanic black, and Mexican American, respectively; P = 0.01 and <0.001) and age of 30–49 years (4.9% vs. 0.9% and 0.8% for non-Hispanic white, non-Hispanic black, and Mexican American, respectively; P = 0.003 and 0.001). IgM seroprevalence also varied significantly by age (5.3%, 7.3%, and 3.7% for women of 12–19, 20–29, and 30–49 years, respectively; P = 0.04) among IgG+ women. Similar to findings from the analysis of CMV IgM among all women, there were significant patterns of decreasing IgM seroprevalence with age in non-Hispanic black and Mexican American women (P<0.001 and = 0.03, respectively), while lack of trend among women of non-Hispanic white (P = 0.37) (Fig 1, Panel B).

The factors previously reported as associated with CMV IgG seroprevalence [5] such as education level, poverty level, insurance, family size, area of residence, census region, or having a child ≤6 years of age at home were not associated with IgM seroprevalence (Table 1). Marital status was associated with IgM seroprevalence among all women aged ≥20 years (4.5% vs. 2.2% for single vs. married women, P = 0.04); however this association was not statistically significant when the analysis was restricted to IgG+ women (6.8% vs. 3.8%, P = 0.11). Association between CMV IgM seroprevalence and sexual behaviors such as number of lifetime sex partners and age at initiation of sexual activity, which were reported as being associated with IgG seroprevalence,[11] could not be examined due to sparsity of the data (approximately 50–60% missing values for these factors).

**Table 1:** Differences in CMV IgM Seroprevalence among Women Aged 12–49 Years by Selected Demographic Factors, NHANES III, 1988–1994.

	All women				IgG+ women			
	IgM+	Sample size	Prevalence (95% CI)	P value	IgM+	Sample size	Prevalence (95% CI)	P value
Overall	121	5714	2.8 (2.1–3.6)	NA	118	4001	4.9 (3.4–6.4)	NA
Race/ethnicity				0.11				<0.001
Non-Hispanic white	52	1825	3.1 (2.0–4.1)		50	892	6.1 (3.8–8.3)	
Non-Hispanic black	44	2013	2.2 (1.5–2.8)		43	1582	2.7 (1.9–3.5)	
Mexican American	25	1876	1.6 (0.6–2.6)		25	1527	2.0 (0.8–3.2)	
Age				0.05				0.04
12–19 years	37	1445	2.4 (1.1–3.6)		37	810	5.3 (2.4–8.2)	
20–29 years	48	1574	4.1 (2.7–5.5)		46	1104	7.3 (4.7–9.9)	
30–49 years	36	2695	2.4 (1.2–3.5)		35	2087	3.7 (1.8–5.7)	
Household income level				0.29				0.48
High	17	990	2.0 (0.5–3.5)		17	516	4.5 (1.1–8.0)	
Middle	54	2185	3.3 (2.4–4.2)		51	1468	5.4 (3.7–7.2)	
Low	37	2070	2.7 (1.7–3.8)		37	1647	4.0 (2.4–5.5)	
Education				0.27				0.78
Completed 11th grade or less	39	2057	3.9 (2.3–5.4)		36	1693	4.6 (2.2–6.9)	
Completed high school	47	1850	3.1 (2.1–4.1)		47	1258	5.5 (3.7–7.2)	
College or higher	34	1767	2.2 (0.9–3.5)		34	1018	4.7 (2.0–7.4)	
Family size				0.19				0.09
1–2	38	1207	3.9 (1.8–6.1)		37	811	6.7 (3.1–10.2)	
3–4	46	2452	2.5 (1.6–3.4)		45	1622	4.9 (3.1–6.7)	
5+	37	2055	2.0 (1.1–3.0)		36	1568	2.9 (1.5–4.3)	
Insurance				0.74				0.88
Government	18	993	2.8 (0.4–5.2)		18	787	4.0 (0.6–7.4)	
Private	79	3567	2.7 (1.8–3.7)		76	2263	5.1 (3.1–7.0)	
Uninsured	24	1154	3.6 (1.5–5.7)		24	951	4.9 (2.0–7.9)	
Area of residence				0.56				0.73
Urban	50	2875	2.5 (1.1–4.0)		50	2074	4.6 (1.9–7.3)	
Non-urban	71	2839	3.1 (2.2–3.9)		68	1927	5.2 (3.6–6.7)	
Census region				0.31				0.28
Northeast	18	609	3.6 (2.3–4.9)		17	373	7.5 (3.4–11.5)	
Midwest	28	1104	3.0 (1.7–4.2)		27	691	5.3 (3.1–7.6)	
South	48	2527	2.9 (1.1–4.6)		47	1850	4.5 (1.8–7.2)	
West	27	1474	1.9 (0.9–2.9)		27	1087	3.4 (1.3–5.4)	
Birthplace				NA				NA
United States	116	4656	3.0 (2.1–3.8)		113	3028	5.3 (3.7–6.9)	
Mexico	3	836	0.3 (0.0–0.6)		3	784	0.3 (0.0–0.7)	
Other country	2	208	2.2 (0.0–5.7)		2	177	2.9 (0.0–7.6)	
Have child aged ≤6 years at home				0.79				0.96
Yes	47	2186	3.0 (1.8–4.2)		47	1694	4.9 (3.0–6.9)	
No	74	3528	2.8 (1.8–3.7)		71	2307	4.9 (2.9–6.8)	
Marital status*				0.04				0.11
Married	46	2713	2.2 (1.6–2.9)		44	1972	3.8 (2.4–5.2)	
Single	38	1550	4.5 (1.7–7.4)		37	1214	6.8 (2.5–11.1)	

NA: not applicable.

* in women aged ≥20 years only.

## Discussion

Using data from NHANES III 1988–1994, we present estimates of CMV IgM seroprevalence by race/ethnicity in U.S. women of reproductive age. As a result of excluding “other or unknown” racial/ethnic groups, our analyses demonstrated a slightly lower rate in overall CMV IgM seroprevalence than previously reported (2.8% vs. 3.0%).[7] This small difference in point estimate of CMV IgM seroprevalence does not change our overall understanding of CMV IgM seroprevalence in the U.S. However, the estimates we present by age and racial/ethnic groups provide useful baseline reference values for future studies to monitor CMV rates of transmission over time and assess the relative contribution of different factors to IgM seroprevalence. In contrast to the prior study on IgM seroprevalence, [7] we found IgM seroprevalence did differ across age groups when collapsing 30–49 year olds into a single age group. Given that 98% of CMV IgM+ women were identified from among IgG+ individuals, future analyses of IgM seroprevalence using NHANES data could be more efficiently conducted by limiting the sample to CMV IgG+ subjects, as that would require testing of fewer participants. An additional benefit of this approach is that variations in IgM seroprevalence would be less likely to be diluted by the inclusion of IgG- participants, as evidenced by the significant differences by age and race/ethnicity observed in current analysis on IgG+ women only.

After primary infection, IgG seropositivity is lifelong and thus CMV IgG seroprevalence reflects the accumulation of primary infections. In contrast, CMV IgM is produced transiently as a result of primary infection and non-primary CMV infection (reinfection with a new strain of CMV or reactivation of a latent strain), and, therefore, can be a marker of recent transmission. CMV IgG seroprevalence is relatively low among non-Hispanic whites. Because a greater proportion are susceptible to primary infections, the higher rate of IgM positivity among white females compared to non-Hispanic black and Mexican American women is not surprising. Important sources for CMV transmission in adulthood are thought to include exposure to young children and sexual activity.[11, 12] In our analysis, these factors were not associated with IgM seropositivity, but this may reflect low statistical power due to the small number of IgM positive participants as well as a dilution of the importance of these factors in face of the combined contribution to overall IgM positivity from non-primary infections in addition to primary infections, in contrast to the sole contribution to IgG positivity from primary infection. Differences in the patterns of IgM and IgG seroprevalence with age suggest non-primary infection may account for a substantial proportion of IgM positivity, especially with increasing age. While factors associated with CMV IgM seroprevalence among those undergoing primary infection or reinfection may be similar to those associated with CMV IgG seroprevalence, factors associated with reactivation of latent infection may be very different. Understanding this will be difficult until there are better tools for differentiating reinfection from reactivation. Further study is needed to identify risk factors associated with reinfection and reactivation and to determine the effectiveness of strategies to prevent infection, particularly during pregnancy.

Our finding of 2.8% IgM seroprevalence in U.S. women of reproductive age is similar to estimates reported from other populations with moderate IgG seroprevalence such as France and Japan (4.1% and 2.5%, respectively).[13, 14] In contrast, populations with higher CMV IgG seroprevalence, such as Turkey and Korea (both above 98%), reported having much lower prevalence of IgM in women (0.2% and 1.3%, respectively), and none of the IgM+ subjects in those studies had low IgG avidity that would be suggestive of recent primary infection.[15, 16] Variations in IgM seroprevalence had been observed in populations with high IgG seroprevalence such as India, China, and Brazil, where similar IgG seroprevalence (98.6%, 95.5%, and 96.4%, respectively) had been reported but IgM seroprevalence varies dramatically (0.07%, 0.5%, and 2.3%, respectively).[17–19] Populations with higher maternal CMV IgG seroprevalence generally have higher rates of congenital CMV infection than populations with moderate IgG seroprevalence such as the U.S. [20, 21] The relative contributions of primary and non-primary maternal CMV infection to rates of congenital CMV infection across different populations are not well understood.

In summary, the patterns of CMV IgM seroprevalence differ by age and race/ethnicity in women of reproductive age in the US, and the differences may be useful for understanding the prevalence of transmission and congenital CMV epidemiology, therefore, potentially helpful in identifying target population for effective prevention efforts to reduce CMV transmission, in addition to providing baseline estimates for temporal monitoring on IgM seroprevalence over time.
